# Alcohol Use Patterns Among Underage Autistic and Non-Autistic Youth

**DOI:** 10.1007/s10803-023-06086-4

**Published:** 2023-09-26

**Authors:** Laura Graham Holmes, Ziming Xuan, Emily Quinn, Reid Caplan, Amelia Sanchez, Peter Wharmby, Calliope Holingue, Sharon Levy, Emily F. Rothman

**Affiliations:** 1 Silberman School of Social Work, CUNY Hunter College, New York, USA; 2 Boston University School of Public Health, Boston, USA; 3 Center for Autism and Related Disorders, Department of Mental Health, Kennedy Krieger Institute, Johns Hopkins Bloomberg School of Public Health, Baltimore, USA; 4 Adolescent Substance Use and Addiction Program, Boston Children’s Hospital, Harvard Medical School, Boston, USA; 5 Department of Occupational Therapy, Boston University, Boston, USA

**Keywords:** Autism, Underage drinking, Alcohol, Alcohol use disorder, Hazardous drinking, Substance use

## Abstract

**Purpose:**

We explored factors predicting repeated or hazardous alcohol use among autistic and non-autistic U.S. youth ages 16 to 20 years.

**Methods:**

Autistic (n = 94) and non-autistic (n = 92) youth completed an online survey. By design, half of each group reported past-year alcohol use. We compared drinking patterns for autistic and non-autistic youth, and within each group between abstinent or infrequent drinkers (0–1 drinking episodes in past year) versus those who drank 2 + times in past year.

**Results:**

Autistic (vs. non-autistic) youth who drank did so less frequently and consumed fewer drinks per occasion. However, 15% of autistic youth who drank in the past year reported heavy episodic drinking and 9.3% screened positive for AUDIT-C hazardous drinking. For autistic youth only, a diagnosis of depression, bullying or exclusion histories were positively associated with drinking 2 + times in the past year. Autistic youth who put more effort into masking autistic traits were less likely to report drinking 2 + times in the past year. As compared to non-autistic youth, autistic participants were less likely to drink for social reasons, to conform, or to enhance experiences, but drank to cope at similar rates.

**Conclusion:**

Repeated and hazardous underage alcohol occur among autistic youth. Targeted prevention programs designed to address the specific drinking profiles of autistic youth are needed.

Although most autistic adolescents spend most of their time in general education classrooms with neurotypical peers (U.S. Department of Education, 2020), less than half are included in school-based substance use prevention education (Graham Holmes et al., 2022). This is problematic. Autistic people experience high rates of anxiety (29%), depression (26%), attention deficit disorders (11%), bipolar disorder (11%), schizophrenia (8%), and five times the rate of suicide attempts compared to the general population (Croen et al., 2015). These psychiatric conditions are risk factors for alcohol use disorders (AUD) and other substance use disorders (SUD). Negative societal responses to autistic people, such as stigma and social exclusion, exacerbate these factors (Hosseinbor et al., 2014; Marel et al., 2019; Vaughn et al., 2010). Many autistic young people are sexual or gender minorities (e.g., lesbian, gay, bisexual, queer, asexual, transgender) (Dewinter et al., 2017; Strang et al., 2018) and sexual and gender minority youth drink alcohol and engage in heavy episodic drinking more frequently than cisgender heterosexual youth due to stigma and factors such as anti-LGBTQ school climate (Coulter et al., 2016; Talley et al., 2014). Autistic young people may use alcohol to cope with the stress of experiencing bullying or social exclusion, for tension or social anxiety reduction, or to fit in with others (Rengit et al., 2016), putting them at greater risk for AUD.

Emerging research with large population-based samples suggests that some autistic people are at increased risk of AUD or SUD. For example, a Swedish population-based matched case-control study (n = 26,986) found that autistic people had double the risk of AUD as neurotypical family members (Butwicka et al., 2017). However, not all autistic people share the same risk for SUD. Co-occurring diagnoses of intellectual disability or ADHD are thought to moderate the relationship between autism and substance misuse. Recent studies showed a lower rate of SUD in autistic people without ADHD, but a higher rate in autistic people with ADHD (Yule et al., 2023). Co-occurring intellectual disability is associated with decreased risk for SUD (Butwicka et al., 2017; Santosh & Mijovic, 2006). Co-occurring ADHD, on the other hand, appears to increase the risk of SUD (Butwicka et al., 2017; Ressel et al., 2020; Solberg et al., 2019) and co-occurs very frequently with autism (Ståhlberg et al., 2010; Yule et al., 2023). One other factor associated with AUD for autistic people is age. Research suggests that autistic youth have a similar risk for alcohol-related disorders during adolescence and young adulthood compared to non-autistic youth (Abdallah et al., 2011), and that the age of onset for SUD is significantly older among autistic people than for ADHD or non-autistic controls (Yule et al., 2023).

Historically, researchers and service providers assumed that excessive alcohol use or AUD were not problems for autistic people. Autism is relatively newly defined—it did not appear in the DSM until 1980—and the earliest conceptualizations of autism reflected only people with “higher needs” (i.e., more apparent traits that heavily impact daily living). Thus, until recently, the autistic people with whom researchers and healthcare providers were most likely to engage were people with small or no social networks, who were highly reliant on parents or caregivers, were rarely unsupervised, and had a tendency to appreciate and follow rules (such as those against adolescent experimentation with substances) (Ramos et al., 2013). These autistic people were thought to be less likely than non-autistic people to use tobacco, nicotine, or misuse substances (including alcohol). However, contemporary research includes a broader array of autistic people, so the observed relationships between autism and substance use have changed. Diagnostic criteria may also have affected how clinicians conceptualized the relationship between autism and substance use. While the International Classification of Diseases (ICD) allowed clinicians to diagnose co-occurring autism and ADHD (Solberg et al., 2019; Ståhlberg et al., 2010), only with the advent of the DSM-5 in 2017 were North American clinicians able to do so. Researchers have speculated that clinicians may not have provided accurate autism diagnoses to autistic people who presented with ADHD and substance use due to the mistaken idea that autistic people are not interested in substance use or do not have problems with substance use, which may have further biased research on alcohol use among autistic young people (Butwicka et al., 2017).

Despite the need to better characterize the association between autism and substance use, no prior study has examined whether underage autistic youth are any more or less likely to consume alcohol than non-autistic youth or explored, quantitatively, reasons for drinking in autistic versus non-autistic youth. This information is important for the development of AUD screening guidelines and for adapting existing evidence-based AUD interventions for autistic youth. Here, we use the term “youth” to refer to older adolescents and young adults (i.e., age 16 to 20 years old). This study investigated alcohol use patterns and motivations in an autistic and non-autistic sample of underage youth. Based on our previous qualitative work with autistic youth (Rothman et al., 2022), we know that some have one drink with family for religious or cultural reasons (e.g., wine at Passover, champagne on New Year’s Eve), or may try alcohol once and then avoid it because they did not enjoy it. Therefore, we compared youth who had no drinks or one episode of drinking in the previous year (abstinent or infrequent drinkers) to those who drank two or more times in the previous year.

We used an online survey to test the following hypotheses. We expected that:

1. Autistic youth would initiate drinking at a later age, would report drinking less frequently, and would have fewer drinks per occasion than non-autistic youth.
2. As compared to abstinent or infrequent autistic drinkers, autistic youth who drank 2 + times in the past year would be older, male, more likely to report psychiatric diagnoses such as ADHD, depression, or anxiety, and more likely to report a history of bullying or exclusion.
3. As compared to abstinent or infrequent autistic drinkers, autistic youth who drank 2 + times in the past year would report feeling more relaxed and socially at ease after consuming alcohol and would be more likely to endorse coping motivations for drinking.

## Methods

Procedures were approved by the Institutional Review Board (IRB) at the senior author’s institution. All participants provided written informed consent and received a $40 gift card for participating.

### Participants

Autistic participants were recruited via social media advertising, databases of prior research participants who consented to be re-contacted, an autism higher education listserv, contact with colleges with autism programs, and the Simons Foundation Powering Autism Research project (SPARK) (The SPARK Consortium, 2018). SPARK is a national autism genotyping project that recruits families from 31 U.S. academic medical centers to participate in research through an online registry. Non-autistic participants were recruited through social media advertising and chain referrals from enrolled participants (Penrod et al., 2003). Eligibility criteria were (1) between 16 and 20 years old, (2) current U.S. resident, (3) able to read and write in English, (4) able to complete a self-report survey without assistance from a parent or caregiver, (5) fit into our targeted recruitment scheme (50% drank alcohol in past 12 months, at least 40% female or non-binary, at least 30% Black, Hispanic, or Multiracial), (6) for autistic participants, provided documentation of autism diagnosis (e.g., assessment report, individualized education plan paperwork). Data were collected between January and July 2021. We used quota sampling to meet our targeted recruitment scheme, which in this case meant that we stopped enrolling youth who had not consumed alcohol in the past 12 months once we reached our quota and continued enrolling youth who had consumed alcohol in the past 12 months until we reached our quota. We kept track of gender and race/ethnicity while recruiting but did not need to change our enrollment strategy based on these characteristics.

All potential participants were screened by phone or text for eligibility. For youth under the age of 18, parents consented before youth were screened. We screened 160 total autistic youth and 133 total non-autistic youth. Of autistic youth screened, 97 were eligible for the survey. Three autistic participants consented but did not complete the survey despite reminders. Of non-autistic youth screened, 92 were eligible and 92 completed the study.

Eligible individuals were given the option to complete the survey online via REDCap or by phone if desired, with 13% of autistic youth preferring to complete the survey by phone. We explained aspects of the survey that might frustrate youth (e.g., that similar questions would be asked multiple times for statistical reasons) and told youth that they could contact us with questions about survey items, which several did. The autistic advisory board pilot tested the survey and reported that it took approximately 20 to 30 min to complete all scales. Participants who did not complete the survey in one sitting were contacted with a reminder to complete the survey. All participants who began the survey completed the survey.

### Measures

#### Alcohol Consumption Questionnaire

Three alcohol consumption questions were adapted from the 2018 National Survey on Drug Use and Health (Substance Abuse and Mental Health Services Administration, 2018). NSDUH self-report substance use questions reportedly have high test-retest reliability, ranging from Cohen’s kappa = 0.82 to 0.93 (Center for Behavioral Health Statistics and Quality, 2018). Participants estimated how many days they had a drink in the past 12 months, with the option to answer in days per week, days per month, or days per year. Next, they reported how old they were the first time they drank a full drink of alcohol. Finally, they were asked on how many days in the past 30 days they drank 4 (if female or non-binary) or 5 (if male) drinks on the same occasion (e.g., within a couple of hours). There are no published standards, yet, for how to categorize non-binary participants in terms of heavy episodic drinking, so we chose a conservative categorization in the group with the lower alcohol threshold. Participants were categorized as abstinent/infrequent drinkers (0–1 drinking episodes in the previous year) or those who drank 2 + times in the past year.

Alcohol Sensitivity Questionnaire (ASQ): (O’Neill et al., 2002). The ASQ is a widely used 15-item measure of sensitivity to the effects of alcohol, completed by participants who had ever had a drink. Participants report if they have ever experienced effects of drinking alcohol (e.g., “Do you ever feel high or ‘buzzed’ after drinking alcohol?”, “Do you ever pass out after drinking alcohol?”). The measure has good construct validity as scores predict subjective responses to a dose of alcohol (Fleming et al., 2016); other psychometrics are not available.

#### The Alcohol Use Disorders Identification Test (AUDIT-C)

(Bush et al., 1998). The AUDIT-C is a 3-item measure of active alcohol use problems. It has high sensitivity (above 90%) for detecting problem drinking (Bradley et al., 1998; Bush et al., 1998; Gordon et al., 2001) and good test-retest reliability (ICC = 0.91) (Jeong et al., 2017). Using a 5-point scale, respondents report frequency of drinking (0 = “never”, 4 = “4 or more times a week”), number of drinks during a typical drinking episode (0 = “1 or 2”, 4 = “10 or more”) and frequency of having six or more drinks on one occasion (0 = “never”, 4 = “daily or almost daily”). The number of points is summed (range = 0–12) with higher scores suggesting hazardous drinking or the potential for alcohol use to affect safety. There are sex-based cutoffs for hazardous drinking or active alcohol use disorders (men = 4 + points, women = 3 + points). Participants who reported non-binary or other gender were scored in the hazardous drinking range if they scored 3 + points (Stanton et al., 2021).

#### The CAGE Questionnaire

(Bush et al., 1987). The CAGE is a 4-item screener for active or lifetime alcohol use problems. It has high test-retest reliability (ICC = 0.80-0.95) and adequate construct validity (0.48-0.70) (Dhalla & Kopec, 2007). Respondents report whether they have ever felt they needed to reduce drinking, others have criticized their drinking, they have felt guilty about drinking, or they ever needed a drink first thing in the morning (0 = “no”, 1 = “yes”). Total sum scores range from 0 to 4, with 2 + points considered clinically significant for possible alcohol use problems.

#### Drinking Motives Questionnaire – Revised (DMQ-R)

(Cooper, 1994). The DMQ-R is a 20-item measure of how frequently a person drinks for four different motives (i.e., enhancement, social, conformity, coping). Considering their lifetime alcohol consumption, participants indicate how often they drank for each reason (e.g., “Because it helps you when you feel depressed or nervous”) on a 5-item Likert-style scale from 1 (“never or almost never”) to 5 (“almost always”). Higher subscale scores mean participants attribute more of their drinking to that motive. The DMQ-R subscales have good to excellent test-retest reliability (e.g., ICC = 0.61-0.78) in young adult samples (Grant et al., 2007).

#### Social Responsiveness Scale, 2nd Edition (SRS-2)

(Constantino & Gruber, 2012). The SRS-2 is a 65-item parent-report (< 18 years of age) or self-report (> 18 years of age) rating scale of autism traits, with items falling into social motivation, cognition, awareness, communication, and restricted or repetitive behavior domains. The measure has well-established psychometric properties described in the manual. SRS-2 Total Standard Scores (T-scores) have a mean of 50 and standard deviation of 10, with higher scores indicating greater presence of autism traits. Parents completed the SRS-2 for autistic participants under 18 years of age.

#### Vulnerability Experiences Quotient (VEQ)

(Griffiths et al., 2019). The VEQ was developed with autistic stakeholders to understand negative life experiences that affect mental health. We selected items from the VEQ about bullying or exclusion: being bullied, left out, having rumors spread about them, being hurt badly on purpose by a peer, or being tricked or pressured into giving someone money or possessions. Response options were “yes”, “no”, “don’t know/can’t recall” or “decline to answer”, with “don’t know” or “decline” being coded as missing. One point was coded for each “yes” response such that the range for this instrument was 0–5, with higher scores indicating more experiences of bullying or exclusion. Psychometric information is not available for these selected items.

#### Flourishing Scale

(Diener et al., 2010). The Flourishing Scale is an 8-item measure of a person’s perceived success in relationships, self-esteem, purpose, and optimism. The Flourishing Scale has good internal consistency (α = 0.87), moderately high one-month test-retest reliability (ICC = 0.71), and shows good convergent validity with other measures of well-being (Diener et al., 2010). An example question is, “I actively contribute to the happiness and well-being of others.” Respondents answer questions on a 7-point Likert-style scale from 1 (“strongly disagree”) to 7 (“strongly agree”). A higher summary score indicates greater psychological well-being (range = 8–56).

#### GAD-7

(Spitzer et al., 2006). The GAD-7 measures generalized anxiety disorder (GAD) using a 7-item scale. The GAD-7 has good reliability (α = 0.92), test-retest reliability (ICC = 0.83), and good criterion, construct, factorial, and procedural validity. People respond to the query, “over the last 2 weeks, how often have you been bothered by the following?” (e.g., “Feeling afraid as if something awful might happen”) with response options 0 (“not at all”), 1 (“several days”), 2 (“over half the days”) or 3 (“nearly every day”). Higher scores (range = 0–21) are strongly associated with functional impairment. GAD symptoms are considered mild (5–9), moderate (10–14) or severe (15–21).

#### UCLA Loneliness Scale

(Russell, 1996). The UCLA Loneliness Scale is a 20-item, Likert-style measure with good internal consistency (α = 0.89 or higher) and test-retest reliability (ICC = 0.73) over a 1-year period, and good convergent and construct validity. Respondents indicate how often a list of statements are descriptive of them, such as “How often do you feel that you lack companionship?” Responses range from 1 (“never”) to 4 (“often”). Higher scores (range = 20–80) indicate greater loneliness.

#### Patient-Reported Outcomes Measurement Information System (PROMIS) Depression Scale and Depressive Symptoms Scale

(Irwin et al., 2012; Pilkonis et al., 2011; Quinn et al., 2014). The PROMIS Depression Scale is a 4-item Likert-style measure of depression symptoms for adults (> 18 years old; range = 4–20). The PROMIS Depressive Symptoms Scale is an 8-item self-report measure of depression symptoms for youth (< 18 years old; range = 8–40). Both versions ask respondents about past week symptoms, with responses ranging from 1 (“never”) to 5 (“always”) and higher scores indicating more symptoms of depression. Reliability estimates for PROMIS Depression measures are high (> 0.90) in general population (Teresi et al., 2016) and autistic samples (Holmes et al., 2020). Extensive psychometrics are available (Cella et al., 2019).

#### The Measure of Adolescent Relationship Harassment and Abuse (MARSHA)

(Rothman et al., 2021). The MARSHA is a 22-item measure of physical, sexual, and psychological adolescent dating abuse behaviors. It has good reliability (subscale α’s = 0.68-0.90) and reflects contemporary forms of abuse (e.g., online harassment, coerced nude selfies). Respondents are asked to think about people that they were dating, hooking up with, or in a romantic relationship within the past year. Questions address the frequency of behaviors that may have been perpetrated on them or by them. Responses were 1 (“0 times”), 2 (“1 to 3 times”), 3 (“4 to 10 times”), 4 (“More than 10 times”) or “decline to answer.” A score of 22 (all “0 times” answers) indicates no presence of dating abuse or unhealthy relationship behavior; >22 indicates the presence of such behavior.

#### The Camouflaging Autistic Traits Questionnaire (CAT-Q)

(Hull et al., 2019). The CAT-Q is a 25-item measure assessing strategies that compensate for or mask social interaction difficulties in interactions with acquaintances. Autistic or non-autistic people can respond. The CAT-Q has acceptable internal consistency (α = 0.94), test-retest reliability (*r* = .77), and convergent validity. There are three subscales: Compensation (strategies used to make up for social and communication difficulties, e.g., practicing facial expressions or body language), Masking (strategies or motivation to present a persona that is less or not at all autistic, e.g., feeling pressure to make eye contact), and Assimilation (strategies or motivation to fit in to uncomfortable social situations, e.g., feeling one is pretending to be normal). Questions use a 7-point Likert-style scale ranging from 1 (“strongly disagree”) to 7 (“strongly agree”). Higher scores (range = 25–175) indicate more camouflaging.

### Analyses

Chi square was used to test for differences between groups (autistic vs. not autistic, abstinent/infrequent vs. repeated drinking) for categorical variables. Fisher’s exact test was used for categorical group comparisons with cell counts less than 5. Two-sample t-test was used to test for group differences in continuous variables. Wilcoxian Rank Sum was used to test for group differences for continuous variables with non-normally distributed data (e.g., number of drinks in previous month, age at first drink). To identify variables associated with repeated drinking for autistic and non-autistic subgroups (separately), bivariate and multivariate logistic regression were used. Variables significant at *p*≤.1 in bivariate analyses were included in multivariate analyses, including demographics such as gender, race/ethnicity, and whether the participant attended college at the time of the survey. Less than 5% of data were missing. Two-sided *p* value less than 0.05 was used to indicate statistical significance.

## Results

### Sample Characteristics

We recruited N = 94 autistic youth and N = 92 non-autistic youth. Sample characteristics are presented in Table 1. Reflecting the broader autistic population, the autistic sample had a higher proportion of cisgender males (61.7% vs. 44.6%, *p* = .010) and a lower proportion of heterosexual individuals (48.9% vs. 66.3%, *p* = .023). By chance, the non-autistic sample had a greater proportion of participants who describe their race as Asian (21.7% vs. 6.4%, *p* = .003) and a lower proportion of White participants (63.0% vs. 85.1%, *p* < .001). Autistic participants were more likely than non-autistic to report ADHD, depression, anxiety, and intellectual disability or learning disorder (Table 1). Autistic participants were more likely to attend a 2-year or 4-year college/university (74.5% vs. 48.9%, *p* < .001), more likely to currently reside with parents (92.6% vs. 60.9%, *p* < .001) and were more likely to report ever having free or reduced-price school meals (44.7% vs. 17.4%, *p* < .001). As compared to the non-autistic participants, autistic participants reported more loneliness, more experiences with bullying and exclusion, and scored lower for measures of flourishing.

**Table 1:** Participant demographics by autism status (N = 186)

		Non-autistic (N = 92)				Autistic (N = 94)						
	All	Abstinent or infrequent	Drank 2 + times in past year	Test		All	Abstinent or infrequent	Drank 2 + times in past year	Test			Non-autistic vs. autistic
	% (n)	% (n)	% (n)		*p*	% (n)	% (n)	% (n)		*p*		*p*
Total	100% (92)	100% (47)	100% (45)			100% (94)	100% (64)	100% (30)				
Gender				F	0.544				F	0.499	C	0.010
Cis-Male	44.6 (41)	48.9 (23)	40.0 (18)			61.7 (58)	59.4 (38)	66.7 (20)				
Cis-Female	52.2 (48)	46.8 (22)	57.8 (26)			30.9 (29)	34.4 (22)	23.3 (7)				
Non-binary, transgender, intersex, genderqueer, other	3.3 (3)	4.3 (2)	2.2 (1)			7.4 (7)	6.3 (4)	10.0 (3)				
Race/Ethnicity												
AI/AN/Native Am.	0.0 (0)	0.0 (0)	0.0 (0)	C	--	2.1 (2)	3.1 (2)	0.0 (0)	F	1.00	F	0.497
Asian	21.7 (20)	25.5 (12)	17.8 (8)	C	0.367	6.4 (6)	6.3 (4)	6.7 (2)	F	1.00	C	0.003
Black or African American	5.4 (5)	8.5 (4)	2.2 (1)	F	0.362	2.1 (2)	1.6 (1)	3.3 (1)	F	0.539	F	0.276
Hispanic or Latino	7.6 (7)	10.6 (5)	4.4 (2)	F	0.435	6.4 (6)	7.8 (5)	3.3 (1)	F	0.661	C	0.743
Native Hawaiian or Other Pac. Is.	0.0 (0)	0.0 (0)	0.0 (0)		--	1.1 (1)	1.6 (1)	0.0 (0)	F	1.00	F	1.00
White	63.0 (58)	53.2 (25)	73.3 (33)	C	0.045	85.1 (80)	87.5 (56)	80.0 (24)	F	0.363	C	< 0.001
Multiracial	3.3 (3)	2.1 (1)	4.4 (2)	F	0.613	7.4 (7)	6.3 (4)	10.0 (3)	F	0.676	F	0.331
Other	1.1 (1)	2.1 (1)	4.4 (2)	F	1.00	0.0 (0)	0.0 (0)	0.0 (0)		--	F	0.495
Sexual orientation				C	0.921				C	0.144	C	0.023
Heterosexual or straight	66.3 (61)	66.0 (31)	66.7 (30)			48.9 (46)	53.1 (34)	40.0 (12)				
LGBAP+	27.2 (25)	27.7 (13)	26.7 (12)			41.5 (39)	35.9 (23)	53.3 (16)				
Ever diagnosed with:												
Intellect. dis/developmental dis.	1.1 (1)	0.0 (0)	2.2 (1)	F	1.00	7.4 (7)	4.7 (3)	13.3 (4)	F	0.187	F	0.028
Attention deficit disorder	12.0 (11)	10.6 (5)	13.3 (6)	C	0.717	54.3 (51)	56.3 (36)	50.0 (15)	C	0.632	C	< 0.001
Learning disorder	3.2 (3)	4.7 (2)	2.2 (1)	F	0.616	12.8 (12)	10.9 (7)	16.7 (5)	F	0.511	C	0.012
Depression	17.4 (16)	12.8 (6)	22.2 (10)	C	0.249	52.1 (49)	45.3 (29)	66.7 (20)	C	0.037	C	< 0.001
Anxiety disorder (incl. OCD)	25.0 (23)	14.9 (7)	35.6 (16)	C	0.018	59.6 (56)	56.3 (36)	66.7 (20)	C	0.253	C	< 0.001
Substance or alcohol disorder	1.1 (1)	2.1 (1)	0.0 (0)	F	1.00	0.0 (0)	0.0 (0)	0.0 (0)		--	F	0.497
Bipolar disorder	2.2 (2)	2.1 (1)	2.2 (1)	F	1.00	0.0 (0)	0.0 (0)	0.0 (0)		--	F	0.497
Eating disorder	4.3 (4)	2.1 (1)	6.7 (3)	F	0.356	5.3 (5)	3.1 (2)	10.0 (3)	F	0.153	F	0.747
Opp. defiant disorder/conduct	2.2 (2)	2.1 (1)	2.2 (1)	F	1.00	6.4 (6)	7.8 (5)	3.3 (1)	F	0.664	F	0.152
Post-traumatic stress disorder	2.2 (2)	2.1 (1)	2.2 (1)	F	1.00	8.5 (8)	4.3 (4)	13.3 (4)	F	0.237	F	0.055
Attends college/university	48.9 (45)	57.4 (27)	40.0 (18)	C	0.094	74.5 (70)	75.0 (48)	73.3 (22)	C	0.712	C	< 0.001
Has spouse or intimate partner	26.1 (24)	17.0 (8)	35.6 (16)	C	0.043	20.2 (19)	21.9 (14)	16.7 (5)	C	0.488	C	0.406
Currently reside with parents	60.9 (56)	66.0 (31)	55.6 (25)	C	0.307	92.6 (87)	93.8 (60)	90.0 (27)	F	0.676	C	< 0.001
Free or reduced-price school meals	17.4 (16)	27.7 (13)	6.7 (3)	C	0.014	44.7 (42)	35.9 (23)	63.3 (19)	C	0.014	C	< 0.001
SRS-2 Levels					--				C	0.798		--
SRS-2: Severe	--	--	--		--	30.9 (29)	28.1 (18)	36.7 (11)				--
SRS-2: Moderate	--	--	--		--	26.6 (25)	28.1 (18)	23.3 (7)				--
SRS-2: Mild	--	--	--		--	17.0 (16)	17.2 (11)	16.7 (5)				--
SRS-2: Normal	--	--	--		--	17.0 (16)	18.8 (12)	13.3 (4)				--
GAD-7 Scale: Severe Anxiety	10.9 (10)	10.6 (5)	11.1 (5)	F	1.00	18.1 (17)	14.1 (9)	26.7 (8)	C	0.359	C	0.062
Dating abuse past year	48.0 (24)	41.2 (7)	51.5 (17)	C	0.488	42.9 (12)	28.6 (4)	57.1 (8)	C	0.127	C	0.662
	Mean (SD)	Mean (SD)	Mean (SD)		*p*	Mean (SD)	Mean (SD)	Mean (SD)		*p*		*p*
Age	17.9 (1.4)	17.6 (1.3)	18.2 (1.4)	W	0.043	17.6 (1.3)	17.6 (1.4)	17.6 (1.3)	W	0.910	W	0.145
SRS-2 Score	--	--	--		--	71 (13)	71 (12)	72 (13)	W	0.582		--
Flourishing Scale	47 (7)	45 (8)	48 (6)	W	0.160	42 (9)	43 (10)	41 (8)	W	0.137	W	0.005
GAD-7 Scale score	7 (5)	6 95)	7 (5)	W	0.613	8 (7)	7 (7)	9 (6)	W	0.190	W	0.404
UCLA Loneliness Score	41 (12)	43 (14)	39 (9)	W	0.284	47 (12)	46 (12)	49 (11)	T	0.357	W	< 0.001
PROMIS Depression T-Score^a^	54.5 (10.3)	54.8 (11.4)	54.1 (9.1)	W	0.873	53.7 (11.1)	53.5 (12.0)	54.2 (9.7)	W	0.963	W	0.860
Vulnerability Experiences Quotient	1 (1)	1 (2)	1 (1)	W	0.413	2 (2)	2 (2)	3 (2)	W	0.021	W	< 0.001
MARSHA Total Score^b^	1.6 (2.5)	1.4 (2.5)	1.8 (2.5)	W	0.532	2.8 (4.2)	1.9 (4.4)	3.7 (4.1)	W	0.213	W	0.741
CAT-Q Compensation	26 (11)	27 (11)	26 (11)	T	0.839	37 (11)	36 (11)	37 (12)	T	0.781	W	< 0.001
CAT-Q Masking	35 (8)	34 (9)	37 (7)	T	0.064	34 (10)	36 (8)	30 (12)	W	0.037	W	0.775
CAT-Q Assimilation	25 (10)	26 (11)	25 (10)	T	0.633	33 (10)	32 (10)	34 (9)	T	0.427	W	< 0.001

*Note.* Gender and sexual orientation variables were collapsed to facilitate analyses given small n’s for some responses. AI/AN/Native Am = American Indian, Alaskan Native or Native American. CAT-Q = Camouflaging Autistic Traits Questionnaire. GAD-7 = Generalized Anxiety Disorder-7. LGBAP + = Lesbian, gay, bisexual, asexual, pansexual, homosexual, or other. MARSHA = Measure of Adolescent Relationship Harassment and Abuse. PROMIS = Patient-Reported Outcomes Measurement Information System. SRS-2 = Social Responsiveness Scale, 2nd Edition. Analyses are denoted in the table as follows: Chi square (C) was used to test for differences between groups (autistic vs. not autistic, abstinent/infrequent vs. repeated drinking) for categorical variables. Fisher’s exact test (F) was used for categorical group comparisons with cell counts less than 5. Two-sample t-test (T) was used for continuous variables. Wilcoxian Rank Sum (W) was used for continuous variables with non-normally distributed data (e.g., number of drinks in previous month, age at first drink). “--” denotes that analyses could not be run for these items because data was not collected or cell sizes were too small

^a^ Adults completed the PROMIS Depression scale and youth under 18 completed the PROMIS Depressive Symptoms scale

^b^ The MARSHA was completed by participants who had an intimate/romantic partner in the past year

### Comparison of Alcohol Use by Autism Status

#### Hypothesis 1

Autistic youth would initiate drinking at a later age, would report drinking less frequently, and would have fewer drinks per occasion than non-autistic youth.

The average age of first drink for autistic youth was 15 years old and for non-autistic youth was 16 years old (*p* = .970; Table 2). Approximately 29% of autistic youth and 64% of non-autistic youth reported having one or more drinks in the past month (not shown, *p* < .01). Autistic participants reported fewer days of drinking in the past 12 months than non-autistic participants (30 days vs. 36 days, *p* = .018), and fewer episodes of heavy episodic drinking in the past month (2 vs. 0, *p* = < 0.001). Autistic participants were also less likely to report some drinking consequences such as ever had a hangover (11% vs. 24%, *p* = .018) or vomited after drinking (5% vs. 25.0%, *p* < .001). Additionally, autistic participants were less likely to have either a positive AUDIT-C for hazardous drinking (9% vs. 27%, *p* = .002) or a CAGE score indicating problematic alcohol use (7% vs. 9%, *NS*). Autistic youth were less likely than non-autistic youth to endorse drinking for social reasons (*p* < .001), to enhance experiences (*p* = .006), to conform with a group (*p* = .008) but were as likely to report drinking to cope (*p* = .167).

**Table 2:** Descriptive statistics on alcohol drinking by group (N = 186)

		Non-autistic (N = 92)							Autistic (N = 94)							
	All	Abstinent or infrequent	Drank 2 + times in past year	Test			All		Abstinent or infrequent		Drank 2 + times in past year	Test				Autistic vs. Non-autistic
	% (n)	% (n)	% (n)		*p*		% (n)		% (n)	% (n)			*p*			*p*
**Total**	100% (92)	100% (47)	100% (45)				100% (94)		100% (64)	100% (30)						
Drinking Frequency																
Any past year drinking	51.1 (47)	6.4 (3)	97.8 (44)	C	< .001		41.5 (39)		18.8 (12)	90.0 (27)		C	< .001		C	0.189
Any past month drinking	36.9 (34)	2.1 (1)	73.3 (33)	F	< .001		16.0 (15)		3.1 (2)	43.3 (13)		F	< .001		C	0.001
Any heavy episodic past month	23.9 (22)	0.0 (0)	48.9 (22)	C	< .001		7.4 (7)		3.1 (2)	16.7 (5)		F	0.032		C	0.002
Drinking Consequences																
Hangover after drinking	23.9 (22)	2.1 (1)	46.7 (21)	F	0.064		10.6 (10)		3.1 (2)	26.7 (8)		F	0.279		C	0.018
Passed out after drinking	1.1 (1)	0.0 (0)	2.2 (1)	F	1.00		2.1 (2)		1.6 (1)	3.3 (1)		F	1.00		F	0.610
Vomited after drinking	25.0 (23)	2.1 (1)	48.9 (22)	F	0.061		5.3 (5)		1.6 (1)	13.3 (4)		F	0.636		C	< .001
Blackout after drinking	9.8 (9)	0.0 (0)	5.0 (9)	C	0.160		5.3 (5)		0.0 (0)	16.7 (5)		F	0.142		C	0.302
Socially at ease after drinking	40.2 (37)	4.3 (2)	77.8 (35)	F	0.002		31.9 (30)		12.5 (8)	73.3 (22)		C	0.012		C	0.242
More relaxed after drinking	43.5 (40)	4.3 (2)	84.4 (38)	F	< .001		33.0 (31)		14.1 (9)	73.3 (22)		C	0.028		C	0.108
Drinking Concerns																
AUDIT-C Positive^a^	27.2 (25)	0.0 (0)	55.6 (25)	C	< .001		9.3 (7)		0.0 (0)	23.3 (7)		F	< .001		C	0.002
CAGE Elevated Score (≥2)	8.7 (8)	0.0 (0)	17.8 (8)	F	0.006		6.8 (5)		0.0 (0)	16.7 (5)		F	0.010		C	0.633
	Mean (SD)	Mean (SD)	Mean (SD)		*p*		Mean (SD)		Mean (SD)	Mean (SD)			*p*			*p*
Drinking Frequency																
Drinking days past year (Mean, SD)^f^	36 (41)	1 (0)	39 (42)	W	0.001		30 (64)		1 (0)	37 (69)		W	< .001		W	0.018
Age at first drink (Mean, SD)	16 (2)	17 (1)	15 (2)	W	0.102		15 (4)		16 (4)	14 (4)		W	0.085		W	0.970
Days of heavy episodic drinking past month (Mean, SD)^b^	2 (4)	0 (0)	3 (5)	W	0.002		0 (2)		0 (1)	1 (2)		W	0.306		W	< .001
Drinking Motivations (DMQ-R)																
Social (Mean, SD)	16 (5)	14 (6)	18 (4)	W	< .001	12 (6)			11 (5)	14 (6)		W	0.020	W		< .001
Coping (Mean, SD)	11 (5)	12 (6)	10 (5)	W	0.236	10 (5)			10 (5)	10 (6)		W	0.638	W		0.167
Enhancement (Mean, SD)	13 (5)	11 (5)	16 (5)	W	< .001	11 (6)			9 (5)	14 (6)		W	0.002	W		0.006
Conformity (Mean, SD)	10 (4)	10 (4)	9 (4)	W	0.151	8 (3)			8 (3)	7 (3)		W	0.302	W		0.008
Drinking Concerns																
AUDIT-C Total Score (Mean, SD)	2 (2)	0 (0)	4 (2)	W	< .001	1 (2)			0 (1)	2 (2)		W	< .001	W		0.095

*Note.* AUDIT-C = Alcohol Use Disorders Identification Test-Concise. CAGE = Cut down, Annoyed, Guilty, Eye-opener. DMQ-R = Drinking Motives Questionnaire, Revised. Abstinent or less frequent/More frequent categories were created using the AUDIT-C and CAGE. Analyses are denoted in the table as follows: Chi square (C) was used to test for differences between groups (autistic vs. not autistic, abstinent/infrequent vs. repeated drinking) for categorical variables. Fisher’s exact test (F) was used for categorical group comparisons with cell counts less than 5. Two-sample t-test (T) was used for continuous variables. Wilcoxian Rank Sum (W) was used for continuous variables with non-normally distributed data (e.g., number of drinks in previous month, age at first drink)

^a^ Non-binary participants were categorized using the more conservative score of ≥ 3

^b^ This item was asked as 4 or 5 drinks based on sex (4 for women, 5 for men). Non-binary participants were categorized using the more conservative number of 4 drinks

### Comparison of Abstinent/Infrequent Drinkers to Youth Who Drank 2 + Times in the Past Year, by Autism Status

#### Hypothesis 2

As compared to abstinent or infrequent autistic drinkers, autistic youth who drank 2 + times in the past year would be older, male, more likely to report psychiatric diagnoses such as ADHD, depression, or anxiety, and more likely to report a history of bullying or exclusion.

Four factors were associated with drinking 2 + times in the past year for the autistic group: lifetime depression diagnosis (*p* = .037), having been bullied or excluded (*p* = .021), lower CAT-Q Masking scores (*p* = .037), and ever having had free or reduced-price school breakfast/lunch (*p* = .014; Table 1). For the non-autistic group, four factors related to drinking 2 + times in the past year were older age (*p* = .043), White race (*p* = .045), lifetime anxiety disorder diagnosis (*p* = .018), and having a spouse or intimate partner (*p* = .043). Ever having had free or reduced-price school breakfast/lunch was associated with being an abstinent/infrequent drinker for non-autistic youth (*p* = .014).

#### Hypothesis 3

As compared to abstinent or infrequent autistic drinkers, autistic youth who drank 2 + times in the past year would report feeling more relaxed and socially at ease after consuming alcohol and would be more likely to endorse coping motivations for drinking.

Table 2 shows descriptive statistics on alcohol drinking for autistic and non-autistic subgroups and, within each subgroup, by status as an abstinent/infrequent drinker versus youth who drank 2 + times in the past year. Youth in both subgroups who drank 2 + times in the past year were more likely to report that they felt more socially at ease and more relaxed after drinking compared to abstinent/infrequent drinkers. In both autistic and non-autistic groups, youth who drank 2 + times in the past year had higher mean scores for the social and enhancement of experiences subscales of the DMQ-R compared to abstinent/infrequent drinkers. However, youth who drank 2 + times in the past year were not more likely to endorse coping as a drinking motivation than abstinent or infrequent drinkers.

### Predicting Repeated Drinking

Table 3 shows bivariate and multivariate models with predictors of having drank 2 + times in the past year for autistic participants and non-autistic participants. For autistic participants, drinking 2 + times in the past year was predicted by higher DMQ-R Social (*p* = .019) and Enhancement (*p* = .002) scores, more VEQ bullying and exclusion experiences (*p* = .025), and lower CAT-Q Masking scores (*p* = .013). Due to high correlations between the DMQ-R Social and Enhancement subscales (autistic group *r* = .771, non-autistic group *r* = .746), we included only the DMQ-R Social subscale in the multivariate models. In the multivariate model for autistic participants, higher VEQ score (*p* = .030) and lower CAT-Q Masking score (*p* = .033) predicted drinking 2 + times in the past year. For non-autistic participants, drinking 2 + times in the past year was predicted by White race (*p* = .048), attending college (*p* = .096), higher scores on the DMQ-R Social (*p* < .001) and Enhancement (*p* < .001) subscales, and higher scores on the UCLA Loneliness scale (*p* = .086) and the CAT-Q Masking subscale (*p* = .067). In the multivariate model for non-autistic participants, White race (*p* = .006) and higher score on the DMQ-R Social subscale (*p* = .007) predicted drinking 2 + times in the past year.

**Table 3:** Logistic regression: predictors of categorization as a more frequent drinker among non-autistic and autistic groups (N = 186)

OR (95% CI)									
	Non-autistic (N = 92)				Autistic (N = 94)				
	Drank 2 + times in past year (Bivariate)	*p*	Drank 2 + times in past year (Multivariate)	*p*	Drank 2 + times in past year (Bivariate)	*P*	Drank 2 + times in past year (Multivariate)	*p*	
Potential Confounders									
Male sex	0.66 (0.29, 1.53)	0.335	--	--	1.65 (0.60, 4.53)	0.328	--	--	
White race	2.42 (1.01, 5.80)	0.048	4.97 (1.60, 15.42)	0.006	0.57 (0.18, 1.83)	0.345	--	--	
Heterosexual	1.05 (0.41, 2.66)	0.921	--	--	0.51 (0.20, 1.27)	0.147	--	--	
ADHD diagnosis	1.26 (0.36, 4.49)	0.718	--	--	0.80 (0.32, 2.01)	0.633	--	--	
Resides w/parents	0.65 (0.28, 1.50)	0.308	--	--	0.60 (0.13, 2.87)	0.522	--	--	
Attends college	2.02 (0.88, 4.65)	0.096	2.87 (0.96, 8.55)	0.059	0.82 (0.28, 2.37)	0.712	--	--	
Drinking Motivations (DMQ-R)									
Social	1.17 (1.07, 1.28)	0.001	1.15 (1.04, 1.28)	0.007	1.10 (1.02, 1.20)	0.019	1.10 (1.00, 1.21)	0.050	
Coping	0.94 (0.86, 1.02)	0.128	--	--	1.01 (0.92, 1.10)	0.872	--	--	
Enhancement	1.22 (1.10, 1.34)	0.000	--	--	1.15 (1.05, 1.26)	0.002	--	--	
Conformity	0.93 (0.85, 1.03)	0.171	--	--	0.93 (0.81, 1.08)	0.335	--	--	
Health & Well-being									
GAD-7	1.01 (0.93, 1.10)	0.783	--	--	1.04 (0.97, 1.12)	0.245	--	--	
GAD-7 Severe anxiety	1.05 (0.28, 3.90)	0.942	--	--	1.66 (0.56, 4.90)	0.362	--	--	
UCLA Loneliness	0.97 (0.93, 1.00)	0.086	0.95 (0.91, 1.00)	0.061	1.02 (0.98, 1.06)	0.353	--	--	
PROMIS Depression^a^	0.99 (0.95, 1.03)	0.739	--	--	1.01 (0.97, 1.05)	0.776	--	--	
VEQ	0.82 (0.61, 1.10)	0.191	--	--	1.40 (1.04, 1.88)	0.025	1.44 (1.04, 2.01)	0.030	
MARSHA^b^	1.08 (0.84, 1.39)	0.556	--	--	1.12 (0.92, 1.37)	0.256	--	--	
Camouflaging Autistic Traits Questionnaire									
Compensation	1.00 (0.96, 1.04)	0.837	--	--	1.01 (0.96, 1.05)	0.778	--	--	
Masking	1.05 (1.00, 1.11)	0.067	1.04 (0.97, 1.12)	0.228	0.94 (0.89, 0.99)	0.013	0.94 (0.89, 0.99)	0.033	
Assimilation	0.99 (0.95, 1.03)	0.629	--	--	1.02 (0.97, 1.07)	0.423	--	--	

*Note.* * p < .05 ** p < .01 *** p < .001. DMQ = Drinking Motives Questionnaire. GAD-7 = Generalized Anxiety Disorder-7. PROMIS = Patient-Reported Outcomes Measurement Information System. SP/CBI = Self-Perception and Cognitive & Behavioral Impairment. VEQ = Vulnerability Experiences Quotient

^a^Adults completed the PROMIS Depression scale and youth under 18 years completed the PROMIS Depressive Symptoms scale

^b^The MARSHA was completed by participants who had an intimate or romantic partner in the past year

## Discussion

This study fills the critical gap in research on alcohol use among autistic young people (Adhia et al., 2020). The purpose of the study was to understand patterns among autistic drinkers and non-drinkers compared to non-autistic youth. Our results suggest that underage autistic youth who drink start around the same age as non-autistic youth but do so less frequently and heavily. However, some autistic youth reported negative drinking consequences or hazardous alcohol use. Adolescence is a unique and dynamic time for brain development. Adolescent alcohol use can affect reward neurocircuitry and corticolimbic structure in ways that lead to less behavioral control over drinking and greater vulnerability to AUD in adulthood (Nixon & McClain, 2010). Thus, prevention and treatment of AUD targeted to adolescents and young adults is important. Tailored strategies may be needed given the difference in alcohol use patterns and motivations among autistic youth. Young adults with autism already face many obstacles to high quality of life and physical and mental health. Timely interventions and effective preventative programming can prevent AUD and lasting consequences in adulthood. Currently, there are no interventions or preventative programming that target the specific needs of autistic adolescents or adults (Arnevik & Helverschou, 2016).

Our first hypothesis was that autistic youth would initiate drinking at a later age, would report drinking less frequently, and would have fewer drinks per occasion than non-autistic youth. Underage autistic youth in this study reported a similar age of first drink as non-autistic youth, but consistent with prior research, they reported fewer days of drinking, lower rates of heavy episodic drinking and fewer consequences related to alcohol use (Rothman et al., 2022). Also consistent with prior research, autistic youth who reported free or reduced-price school meals, a proxy for lower socioeconomic status, were more likely to report drinking two or more times in the past year (Najman et al., 2010; Poulton et al., 2002; Reinherz et al., 2000). In this study, 9% of autistic participants (18% who drank in the past year) were positive for hazardous drinking on the AUDIT-C. This is comparable to the rate of hazardous drinkers in a recent survey of autistic adults (Bowri et al., 2021), suggesting the proportion of autistic hazardous drinkers could remain stable over the lifespan.

Our second hypothesis was that autistic youth who drank 2 + times in the past year would be older, male, more likely to report psychiatric diagnoses, and more likely to report a history of bullying or exclusion. In previous research, any drinking, heavy drinking, and frequent heavy drinking increase with age for underage male and female drinkers, with sex-related patterns emerging later in the 18- to 20 year-old and 21- to 25 year-old age ranges (Flewelling et al., 2004). In this study, age and gender did not significantly predict drinking 2 + times in the past year for autistic youth, although age was associated with drinking 2 + times in the past year for non-autistic youth. This may reflect the conceptualization of this variable (i.e., drinking 2 + times in the past year) compared to variables such as any drinking or heavy/frequent heavy drinking among underage youth. Depression was the only psychiatric diagnosis associated with drinking 2 + times in the past year for autistic youth. Depression is highly prevalent among autistic people, affecting an estimated 23–37% (Hollocks et al., 2019). Co-occurring alcohol use and depression are risk factors for suicide (Schneider, 2009), and autistic people have 5 times the odds of attempted suicide (Croen et al., 2015) and 13 times the odds of suicide versus non-autistic people (Hirvikoski et al., 2020). In this study, depression was associated with drinking multiple times in the past year among underage autistic youth, signaling an urgent need for prevention among these youth.

Having experienced bullying and exclusion was related to drinking 2 + times in the past year for autistic youth. Consistent with other research, autistic participants in this study reported more bullying or exclusion experiences growing up versus non-autistic participants (Trundle et al., 2022). Autistic youth who drank 2 + times in the past year had almost two times the odds of having been bullied compared to abstinent/infrequent drinkers. This aligns with research showing bullying victimization is associated with alcohol-related problems, even decades later (Takizawa et al., 2014). Exposure to stress is associated with increased use of alcohol, with some studies highlighting episodic heavy drinking as an avoidant coping mechanism (Topper et al., 2011). In line with this, autistic adults have cited managing trauma as one reason for substance use (Weir et al., 2021).

Our third hypothesis was that autistic youth who drank 2 + times in the past year would report feeling more relaxed and socially at ease after consuming alcohol and would be more likely to endorse coping motivations for drinking compared to abstinent or infrequent drinkers. Among youth in this sample who ever had a drink, those two drank 2 + times in the past year were more likely to report feeling more socially at ease or relaxed after drinking alcohol compared to other youth who ever had a drink (non-drinkers were not asked this question). This aligns with previous research suggesting that autistic youth may use drinking as a tool to feel less socially inhibited and more authentic, more socially motivated, and to cope with stress (Rothman et al., 2022). Alcohol is widely used as a social lubricant and relaxant, and this is not necessarily a problem unless alcohol is a person’s primary or only coping mechanism or they have difficulty controlling their drinking. Research suggests this is an issue for some autistic people – for example, autistic people with social or generalized anxiety are more likely to report hazardous drinking patterns (Bowri et al., 2021). Thus, improving coping skills for socializing and relaxing may be a useful prevention target.

In contrast to previous research (Dixon et al., 2009), coping-related drinking motivations did not distinguish youth who were abstinent/infrequent drinkers versus those who drank 2 + times in the past year. However, it is possible that individuals who begin drinking frequently as underage youth may increasingly use alcohol to cope and worsen risk for AUD as in adulthood. Autistic youth were also less likely to report social enhancement, or conformity (e.g., because your friends pressure you to drink, to fit in with a group you like) motivations to drink compared to non-autistic youth. This suggests that AUD prevention efforts aimed at autistic youth may need to target messaging toward a different, relevant motivations. Qualitative research suggests these may include a focus on physical and mental health effects of alcohol, increased vulnerability to victimization, and risk for dependence or addiction (Rothman et al., 2022). It may also be helpful to provide other ways of achieving goals that people use alcohol for, such as coping with distress, managing sleep issues, or feeling more relaxed during social engagements (Weir et al., 2021).

Autistic youth who reported *less* masking of their autistic traits were more likely to report drinking 2 + times in the past year. This aligns with prior research showing that some autistic youth do not drink in order to avoid “doing something embarrassing” or forgetting to mask (Rothman et al., 2022). Autistic people who are not concerned about masking may be willing to drink more frequently or heavily. The only previous study on alcohol use and social camouflaging did not find a relationship between the total CAT-Q score and AUDIT score for autistic adults (Bowri et al., 2021). The relationship between masking autistic traits and drinking could change with age or accumulated stress/exhaustion. It is also notable that we only found a relationship between more frequent or heavy drinking and the CAT-Q Masking subscale, not CAT-Q total score or other subscales. A nuanced investigation of the related concepts of social camouflaging and masking using a life course developmental approach could shed light on their association with alcohol.

In this study, autistic youth were more likely than non-autistic youth to report that they were transgender or sexual minorities, consistent with previous autism research studies (Dewinter et al., 2017; Kallitsounaki & Williams, 2022). Although larger percentages of autistic youth who were sexual or gender minorities reported drinking 2 + times in the past year than did cisgender or heterosexual youth, these results were not statistically significant. It is important to note that the sample size for this study limited the ability to stratify by demographic variables. Researchers may consider using medical record data or large national datasets such as the ABCD dataset to investigate alcohol and substance use patterns among sexual and gender minority autistic youth and adults (Adhia et al., 2020).

### Limitations

The findings for this cross-sectional study are associational and not causal. Prospective longitudinal research would further assess risk factors for AUD or SUD for autistic people. Additionally, due to the design (recruiting half drinkers and half non-drinkers, convenience sample), we are not able to estimate prevalence of underage drinking in autism; a future study could address this issue. The sample size for some of these analyses was low, and it will be important for these results to be repeated with a larger sample in the future. Next, the demographic composition of the autistic group was different than the non-autistic group (i.e., in the autistic group, more cisgender men, more White people, more participants attending college or university versus high school, and more psychiatric conditions), which could impact generalizability. Underage alcohol use patterns can vary by ethnicity and race (Terry-McElrath & Patrick, 2020), and this study included primarily White and Asian youth. Future research with more racially diverse autistic people would allow for stratification and understanding about how intersecting identities affect risk for AUD. We did not collect information about whether youth were prescribed psychotropic medication. Given the prevalence of such medication use among autistic people (Coury et al., 2012; Esbensen et al., 2009) and potential interactions with alcohol, which some youth have described as a preventative factor in their decision not to drink (Rothman et al., 2022), this would be a useful avenue for future research. Finally, this study focused on autistic youth who could complete a survey independently, and therefore does not generalize to all autistic youth.

## Conclusions

Our results suggest that autistic youth may have different factors that motivate their drinking compared to non-autistic youth, and different demographic factors or experiences that influence them to drink repeatedly or heavily. This suggests that current programming developed for neurotypical young people may not be effective for autistic youth. Given how detrimental hazardous drinking and AUD are to health and quality of life, the development of autism-specific SUD prevention and intervention strategies are urgently needed.
